# The NLR family pyrin domain containing 3 inflammasome in the mechanism of electroacupuncture: Current status and future perspectives

**DOI:** 10.3389/fnagi.2022.913881

**Published:** 2022-10-19

**Authors:** Min Yuan, Dong Wang, Jiaen Yang, Hai Lan

**Affiliations:** ^1^Department of Rehabilitation Medicine, Affiliated Hospital and Clinical Medical College of Chengdu University, Chengdu, China; ^2^Department of TCM Rehabilitation Medicine, Affiliated Foshan Gaoming Hospital of Guangdong Medical University, Foshan, China; ^3^School of Automation Engineering, University of Electronic Science and Technology of China, Chengdu, Sichuan, China

**Keywords:** NLRP3 inflammasome, electroacupuncture, mechanism, diseases, treatment

## Abstract

Electroacupuncture, which is the most widely used alternative medicine treatment, has been gradually recognized for its effectiveness; however, its mechanism of action is not fully understood. The NLR family pyrin domain containing 3 (NLRP3) inflammasome is a thoroughly studied inflammasome that is closely associated with Alzheimer’s disease, spinal cord injury, and other diseases and plays an important role in the diagnosis and treatment of human immune system diseases. In recent years, some scholars have found that the NLRP3 inflammasome is a part of the mechanism of action of electroacupuncture, which has attracted further attention. In the current review, using “electroacupuncture” and “NLRP3 inflammasome” as keywords and based on the existing randomized controlled trials or clinical trials, we summarize the mechanisms of electroacupuncture targeting NLRP3 inflammasome in the treatment of different diseases and discuss how to optimize the electroacupuncture protocol to obtain thorough mechanisms of NLRP3 inflammasome in electroacupuncture and improve the level of evidence.

## Introduction

Electroacupuncture is a kind of therapy that prevents and treats diseases by passing a trace current close to human bioelectricity through a needle after acquiring qi at the acupuncture point. Electroacupuncture, which is a recognized alternative medicine, has been widely used to treat various diseases by reducing inflammation (Zhu et al., 2015; Meng et al., 2018; Shi et al., 2020; Xie et al., 2021). For example, electroacupuncture relieves pain in patients with knee osteoarthritis, partly by reducing pro-inflammatory factors tumor necrosis factor-alpha (TNF-α) and Interleukin 1 beta (IL-1β) (Shi et al., 2020). And electroacupuncture has a protective effect on intestinal function by inhibiting the progression of inflammatory reactions through the reduction of procalcitonin (PCT) and TNF-α in patients with sepsis (Meng et al., 2018). Scholars have conducted various studies to identify its internal mechanism (Lee et al., 2017; Zhang et al., 2017, 2020a); however, a widely recognized unified theory has not yet been formed. In recent years, many scholars have focused on exploring the mechanism of electroacupuncture by targeting the NLRP3 inflammasome.

The definition of inflammasome was first proposed in 2002 (Martinon et al., 2002), and the NLR family pyrin domain containing 3 (NLRP3) inflammasome is one of the most thoroughly studied. NLRP3 inflammasome is a protein complex that mainly exists in the cytoplasm and is formed by the sensor protein NLRP3, the adaptor protein apoptosis-associated speck-like protein containing a CARD (ASC), and the effector protein caspase-1 (Swanson et al., 2019). NLRP3 is inactive in its resting state and can only form an inflammasome when activated. Specific activators are pathogen-associated molecular patterns (PAMPs), host-derived risk signals (danger-associated molecular patterns, DAMPs), and environmental stimuli (Schroder and Tschopp, 2010). When PAMPs or DAMPs are recognized by Toll-like receptors (the first signal), they activate the transcription factor nuclear factor-kappa B (NF-κB) and initiate the transcription of NLRP3, pro-interleukin (IL)-1β, and pro-IL-18, upregulating the expression level of the inflammasome (Zhao and Zhao, 2020). The second signal [adenosine triphosphate (ATP), some bacterial toxins or particulate matter] mediates NLRP3 oligomerization, assembling NLRP3, ASC, and caspase-1 precursor proteins into a complex that induces caspase-1 activation and the secretion of IL-1β and IL-18 (Wu et al., 2018), which promotes the release of inflammatory factors and exacerbates the inflammatory response. To date, the NLRP3 inflammasome, which is an important component of innate immunity, plays an important role in the immune response and disease occurrence (Kelley et al., 2019). It has been reported that NLRP3 inflammasome activation may play an important role in the pathogenesis of inflammatory bowel disease (Zhen and Zhang, 2019), stroke (Alishahi et al., 2019; Sun et al., 2019), Alzheimer’s disease (Bai and Zhang, 2021), and cardiovascular disease (Olsen et al., 2022).

Increasing evidence primarily from animal studies suggests that the NLRP3 signaling pathway may be part of the mechanism by which electroacupuncture can treat various diseases, such as inflammatory bowel disease (Zeng et al., 2018; Song et al., 2019), stroke (Deng et al., 2021), Alzheimer’s disease (He et al., 2020), ischemic heart disease (Zhang et al., 2020c), and inflammatory pain (Yu et al., 2020). However, the underlying mechanism is not fully clear, and the evidence needs to be confirmed through more high-quality studies.

In this study, we have summarized the existing evidence that electroacupuncture inhibits NLRP3 inflammasome from different pathways such as ionic flux, mitochondrial dysfunction, the production of reactive oxygen species, and lysosomal damage, to treat different inflammatory-related diseases, including digestive system disease, neurological disease, circulatory system disease, reproductive system disease, rheumatic immune system disease, and other diseases. In addition, we briefly discuss the protocol quality problems and several factors that would help optimize the electroacupuncture protocols to improve treatment outcomes when applied in future clinical situations.

## Methods

To identify all studies that explored electroacupuncture mechanisms targeting the NLRP3 inflammasome, we performed an electronic literature search in four databases, including Google Scholar, PubMed, the China National Knowledge Infrastructure (CNKI), and WanFang Data Information Site. Search terms were “electroacupuncture” or “EA” or “acupuncture” and “NLRP3” or “NLRP3 inflammasome.” The search was restricted to English or Chinese language articles and included both randomized controlled trials and clinical trials. Relevant literature was searched from 1 January 2011 to 31 March 2022. Eligibility evaluation was done by title and abstract reviews and when abstracts did not provide enough information, the full text of the paper was retrieved for evaluation. If data were duplicated and had been published more than once, the comprehensive study was chosen for inclusion in the review.

## Mechanism of electroacupuncture targeting NLR family pyrin domain containing 3 inflammasome in the treatment of different diseases

Currently, some studies have shown that electroacupuncture can alleviate diseases by targeting the NLRP3 inflammasome (Zeng et al., 2018; Song et al., 2019; He et al., 2020; Deng et al., 2021). Therefore, in this article, we have summarized the reported mechanisms of electroacupuncture targeting NLRP3 inflammasome in different diseases.

### Digestive system diseases

Inflammatory bowel disease (IBD) is a group of chronic non-specific inflammatory intestinal diseases, including ulcerative colitis (UC) and Crohn’s disease (CD). The pathogenesis of UC is not completely understood, but we know that immune factors play an important role in the pathogenesis of UC (Cui and Sun, 2019). As an important part of innate immunity, NLRP3 plays dual roles by promoting inflammation and maintaining intestinal homeostasis (Gorfu et al., 2014). It has been reported that electroacupuncture can reduce the inflammatory development of UC and the symptoms of abdominal pain and diarrhea (Wu and Liang, 2018; Liu H. et al., 2021). In dextran sulfate sodium (DSS)-induced UC, electroacupuncture stimulation at Zusanli (ST36) controlled the balance of proinflammatory and anti-inflammatory factors by inhibiting M1 macrophages and promoting the polarization of M2 macrophages, inhibited NLRP3 inflammation activation, and promoted the protein expression of nuclear factor erythroid 2-related factor (Nrf2) and heme oxygenase 1 (HO-1), thus ameliorating UC (Song et al., 2019). Additionally, reactive oxygen species (ROS) production may be a critical upstream event for the activation of the NLRP3 inflammasome (Zhen and Zhang, 2019). Zeng and colleagues reported that in UC, electroacupuncture stimulation at Qihai (RN6) and bilateral Tianshu (ST25) could treat UC by inhibiting the activation of nicotinamide adenine dinucleotide phosphate oxidase (NOX), reducing the production of ROS, down-regulating the expression of the NLRP3 inflammasome, and reducing the release of pro-inflammatory factors (Zeng et al., 2018).

Non-alcoholic fatty liver disease (NAFLD) is the most common cause of chronic liver disease in Europe. The pathogenesis of NAFLD is complex. In recent years, it is believed that IL-18 and other cytokines can induce insulin resistance by affecting insulin signaling, leading to the occurrence and development of NAFLD (Zhou and Wei, 2018). Currently, there are no drugs to treat this disease clinically, and the main treatment is lifestyle change and symptomatic treatment (Weiß et al., 2014). Electroacupuncture can regulate blood lipid and liver function and has a certain benign regulatory effect on fatty liver, and its mechanism is related to the downregulation of toll-like receptor 4 (TLR4) and Nuclear factor-kappa B (NF-κB) in liver tissue (Chen et al., 2014). Ma and colleagues indicated that electroacupuncture stimulation at Fenglong (ST40), Yinlingquan (SP9), and Sanyinjiao (SP6) decreased the levels of p-NF-κB p65, p-IκBα, p-IKKα, and p-IKKβ by increasing the expression of Sirt1 and inhibiting the NLRP3/NF-κB signaling pathway and inflammation levels, thereby alleviating liver injury (Ma et al., 2020). The detailed experimental protocols are listed in Table 1.

**TABLE 1:** Mechanism of electroacupuncture targeting NLRP3 inflammasome in the treatment of ulcerative colitis and non-alcoholic fatty liver disease.

Disease	Authors	Research object	Acupoint	Time	Frequency	Evaluation	Mechanism
Ulcerative colitis	Song et al., 2019	64 mice	ST36	Qd, 30 min/time, continuous for 6 days	2 groups: low: 10 Hz; high: 100 Hz	Calculate daily body weight, stool consistency, and rectal bleeding. Evaluation of DAI. TNF-α, IL-6, IL-10, IL-12, and IL-1β. Macrophage subgroup.	EA inhibits the expression levels of NLRP3 and IL-1β. High-frequency EA can upregulate Nrf2/HO-1 pathway
	Zeng et al., 2018	40 mice	ST25, and RN6	Qd, 20 min/time, continuous for 14 days	Rarefaction-dense wave	General condition of mice: weight, mental state, diet, and water. Evaluation of DAI. Evaluation of CMDI. The pathological tissue of colon. NLRP3, NOX, ROS, and IL-1β in blood and NLRP3 mRNA and IL-1β mRNA in colon tissue.	EA inhibits the NOX/ROS/NLRP3 pathway (oxidative stress)
Non-alcoholic fatty liver disease	Ma et al., 2020	30 mice	ST40, SP9, and SP6	Treatment for 2 weeks	Not indicated	Measure the levels of ALT, AST, TC, and TG. Measure the levels of serum and liver inflammatory cytokines IL-1β, TNF-α, and IL-6.	EA enhances the expression of Sirt1, and inhibits the NF-κB/NLRP3 pathway

ALT, Alanine aminotransferase; AST, Aspartate aminotransferase; CMDI, Colon mucosa damage index; DAI, Disease activity index; EA, Electroacupuncture; IL, Interleukin; NLRP3, NLR family pyrin domain containing 3; TNF-α, Tumor necrosis factor alpha; NF-κB, Nuclear factor-kappa B; NOX, nicotinamide adenine dinucleotide phosphate oxidase; ROS, Reactive oxygen species; Nrf2/HO-1, Nuclear factor erythroid 2-related factor/Hem oxygenase 1; TC, Total plasma cholesterol; TG, Triglyceride.

### Neurological diseases

The research on acupuncture in the treatment of neurological diseases mainly focused on stroke, Alzheimer’s disease (AD), and spinal cord injury.

Stroke is a sudden disorder of cerebral blood circulation that is divided into ischemic stroke and hemorrhagic stroke. In ischemic stroke, NLRP3 expression and activation in microglia can be rapidly induced by DAMPs to accelerate neurotoxin production and rapidly change neuronal activity and synaptic function, thereby exacerbating the condition (Ma and Wang, 2019). A previous study has proved that neuronal a7 nicotinic acetylcholine receptor (α7nAChR) plays a role in the reduction of post-ischemic neuroinflammation by electroacupuncture (Wang et al., 2012), and Jiang and colleagues further revealed the underlying mechanism that electroacupuncture can inhibit the expression of NLRP3 inflammasome and reduce post-ischemic neuroinflammation by upregulation of α7nAChR (Jiang et al., 2019). In addition, electroacupuncture stimulation of Waiguan (TE5) and Zusanli (ST36) regulated the miR-223/NLRP3 pathway (Sha et al., 2019) and improved the inflammatory response in rats with ischemic stroke. In stroke rat models, electroacupuncture stimulation at Yanglingquan (GB34), Guanyuan (RN4), and Zhaohai (KI6) + Shenmai (BL62) may inhibit the protein expression of NLRP3, caspase-1, and procaspase-1, further inhibit microglia and the number of TUNEL-positive cells (decrease neuronal apoptosis), reduce NLRP3/caspase-1-mediated damage to microglia and neurons (Deng et al., 2021) and improve neurological functional defects associated with stroke. Based on research in a hemorrhagic stroke model in rats, the nerve inflammatory reaction induced by hemorrhagic stroke can induce the release of a variety of inflammatory cytokines, activate NLRP3-mediated inflammation, and accelerate inflammatory cell apoptosis. However, acupuncture stimulation of Baihui (GV20) through Qubin (GB7) can inhibit the expression of NLRP3, inhibit inflammatory reactions, and promote neuronal functional recovery in rats (Liu H. et al., 2020).

Alzheimer’s disease is a neurodegenerative disease with a complex pathogenesis. However, studies have shown that the neuroinflammatory response generated by aging is an important factor in the pathogenesis of AD (Hane et al., 2017; Li et al., 2021), and this neuroinflammatory response activates microglia and releases inflammatory mediators through the NLRP3 inflammasome, such as IL-1β and IL-18, exacerbating the inflammatory response and leading to nerve cell necrosis and cognitive dysfunction (Fu et al., 2017). Therefore, various studies have demonstrated that inhibiting NLRP3 inflammasome-mediated inflammation may be a potential strategy for the treatment of AD. The results of electroacupuncture stimulation at Baihui (GV20) and Zusanli (ST36) showed that electroacupuncture might prevent and treat AD by reducing the protein expression of NLRP3, caspase-1, and IL-1β and the number of activated microglia in the brains of AD model rats (He et al., 2020). Jiang and colleagues reported a consistent effect of electroacupuncture stimulation of AD model rats at Baihui (GV20), Yintang (EX-HN3), and Shuigou (DU26) (Jiang et al., 2018). Consistent with this result, electroacupuncture stimulation at Dazhui (DU-14), Baihui (GV20), Shenshu (BL23), and Zusanli (ST36) improved the cognitive function of AD rats by inhibiting the NLRP3/ASC/Caspase-1 signaling pathway (Zhao et al., 2020). Electroacupuncture stimulation at Baihui (GV20) and Shenting (DU24) inhibited the activation of the NLRP3 inflammasome, thus improving the cognitive deficits associated with AD (Hou et al., 2020). Electroacupuncture stimulation at Baihui (GV20) and Zusanli (ST36) at different frequencies inhibited NLRP3-induced pyroptosis, and compared with 2 Hz, 10 Hz electroacupuncture significantly reduced the expression levels of inflammatory factors, such as IL-1β, IL-6, IL-18, and tumor necrosis factor alpha (TNF-α), thereby improving cognitive function (Tian et al., 2021).

Spinal cord injury (SCI) refers to the partial or complete loss of motor, sensory, autonomic and reflex functions below the injury stage after SCI. Electroacupuncture is effective in the treatment of SCI, which may be related to the inhibition of inflammatory response, the inhibition of apoptosis-related factors and the promotion of neuronal axon regeneration (Li et al., 2019). It is noteworthy that the inflammasome plays an important role in the process of pyroptosis by interacting with upstream signaling molecules in the pyroptosis signaling pathway and then transmitting signals to downstream proteins through activated caspase-1, resulting in cell rupture and the release of IL-1β and IL-18 (Liu L. et al., 2021). It has been suggested that electroacupuncture stimulation at Jiaji (EX-B2) may inhibit upstream P2X7 receptor (P2X7R) expression of the NLRP3 inflammasome, thereby blocking NLRP3 activation and reducing the release of inflammatory factors (Guo et al., 2021; Mei et al., 2021). Another study showed that electroacupuncture stimulation at Dumai (DU), Zhiyang (DU9), and Jizhong (DU6) upregulated the expression of the upstream factor calcitonin gene-related peptide (CGRP), which located in the anterior horn of the spinal cord, inhibited activation of the NLRP3 inflammasome and inhibited the protein expression of NLRP3/ASC/caspase-1, thus playing a role in the treatment of SCI (Wenger et al., 2010). Therefore, P2X7R/NLRP3 and CGRP/NLRP3 may be new therapeutic targets for SCI treatment. The detailed experimental protocols are listed in Table 2.

**TABLE 2:** Mechanism of electroacupuncture targeting NLRP3 inflammasome in the treatment of stroke, Alzheimer’s disease and spinal cord injury.

Disease	Authors	Research object	Acupoints	Time	Frequency	Evaluation	Mechanism
Stroke	Jiang et al., 2019	Adult male Sprague-Dawley rats (quantity not indicated)	GV20	Qd, 30 min/time, continuous for 5 days	2/15 Hz, 1 mA	Evaluate neurobehavioral score and measure infarct volume. Evaluate apoptotic neuronal death. Measure the levels of IL-18, TNF-α, TGF-β1, IL-10, and NLRP3.	EA stimuli induces the a7nAChR-dependent regulation of NLRP3 inflammasome
	Sha et al., 2019	25 mice	TE5 and ST36	Qd, 30 min/time, continuous for 7 days	Continuous wave (20 Hz, 1 mA)	Evaluate the neurological severity score. Infarct volume and brain water content measurements. Evaluate apoptotic neuronal death. Measure the levels of miR-223, NLRP3, caspase-1, IL-18, and IL-1β.	EA alleviates neuroinflammation by inhibiting the miR-223/LRP3 pathway
	Deng et al., 2021	48 mice	Group 1: GB34 + adjunct acupuncture points Group 2: RN4 Group 3: KI6 + BL62	Qd, 30 min/time, continuous for 14 days	Rarefaction wave, 2/100 Hz	Longa score. Detect the Microglia, neuronal morphology, and apoptosis. Measure the expression of NLRP3, caspase-1, and pro-caspase-1.	EA inhibits the expression of NLRP3, caspase-1, and pro-caspase-1 in brain tissue
Alzheimer’s disease	He et al., 2020	36 mice	GV20 and ST36	Qd, 20 min/time, continuous for 8 weeks	Continuous wave (50 Hz, 1 mA)	Morris water maze experiment. Measure the expression of NLRP3, Caspase-1, IL-1β. Measure the number of microglia.	EA inhibits the activation of NLRP3 inflammasome, and down-regulates the expression of caspase-1 and IL-1β
	Zhao et al., 2020	46 mice	DU-14, GV20, BL23, and ST36	Qd, 20 min/time, 2 subgroups: 7 and 21 days	Rarefaction-dense wave (2/15 Hz, 2 mA)	Water maze, new object recognition and platform jumping experiment Measure the expression of NLRP3, ASC, and caspase-1.	EA inhibits the expression of NLRP3, ASC, and caspase-1
Spinal cord injury	Li et al., 2019	36 mice	Two pairs of EX-B2, T9, and T11	Qd, 30 min/time, 2 subgroups: 3 and 7 days	Current ranges from 0.4 to 0.6 mA	Evaluate the BBB. Measure the expression of NLRP3, ASC, cleaved-caspase-1.	EA inhibits NLRP3 inflammasome overactivation and reduces caspase-1 expression
	Liu L. et al., 2021	120 mice	Two pairs of EX-B2, T9, and T11	Qd, 30 min/time, 4 subgroups: 1, 3, 7, and 21 days	100 Hz, 1–2 mA	Evaluate the BBB. Measure the expression of NLRP3 mRNA. Measure the expression of NLRP3, P2X7R, and OX42.	EA inhibits the ATP-P2X7R-NLRP3 pathway
	Mei et al., 2021	72 mice	Two pairs of EX-B2, T9, and T11	Qd, 30 min/time, 4 subgroups: 1, 3, 7, and 21 days	100 Hz	Evaluate the BBB. Measure the expression of NLRP3 and P2X7R mRNA.	EA inhibits the expression of P2X7R, thus reducing the expression NLRP3
	Guo et al., 2021	36 mice	DU9 and DU6	Qd, 30 min/time	Continuous wave, 2 Hz, the intensity of current was measured by the slight tremor of the hind limb	Evaluate the BBB. Measure the expression of CGRP, NLRP3, ASC and caspase-1.	EA upregulates the expression of CGRP and down-regulates the expression of NLRP3, ASC, and caspase-1

ASC, Apoptosis-associated speck-like protein; ATP, adenosine triphosphate; a7nAChR, a7 nicotinic acetylcholine receptor; BBB, Basso-Beattie-Bresnahan; EA, Electroacupuncture; TGF-β1, Transforming growth factor-β1; CGRP, Calcitonin Gene-related Peptide; IL, Interleukin; NLRP3, NLR family pyrin domain containing 3; P2X7R, P2X7 receptor; TNF-α, Tumor Necrosis Factor Alpha.

### Circulatory system diseases

Ischemic heart disease (IHD), which is also known as coronary heart disease (CHD), is caused by narrowing of the coronary arteries that supply blood to the heart muscle (Wenger et al., 2010). Myocardial ischemia leads to the stimulation of macrophages by a large number of necrotic cardiomyocytes, which in turn activates high expression of NLRP3 and promotes an excessive inflammatory response (Gomez et al., 2018), exacerbating myocardial ischemic injury. On the one hand, acupuncture preconditioning can protect cardiovascular function in the early stage of myocardial ischemia (Li et al., 2013). Research has shown that electroacupuncture stimulation at bilateral Neiguan (PC6) protects the myocardium from ischemic injury, possibly by reducing the number of macrophages in the spleen and heart and inhibiting the expression of NLRP3, thus inhibiting the inflammatory response in the myocardium (Zhang et al., 2020b,c). On the other hand, a previous study has demonstrated that NF-κB, as an upstream signal, participated in the regulation of NLRP3 inflammasome expression in the treatment of IHD, thereby improving cardiac function in rats with myocardial ischemia-reperfusion injury and reducing the extent of myocardial infarction (Wang et al., 2021). Meanwhile, Cai and colleagues reported that electroacupuncture postconditioning stimulation at Neiguan (PC6) and Xinshu (BL15) decreased the expression of IL-1β by inhibiting NF-κB signal pathway and decreased the ischemic area and infarct size of rat myocardium (Cai et al., 2014). This mechanism may be related to the downregulation of NLRP3 inflammasome expression by inhibiting NF-κB signal pathway and then decreasing the expression of IL-1β and other inflammatory factors. The detailed experimental protocols are listed in Table 3.

**TABLE 3:** Mechanism of electroacupuncture targeting NLRP3 inflammasome in the treatment of ischemic heart disease.

Disease	Authors	Research object	Acupoints	Time	Frequency	Evaluation	Mechanism
Ischemic heart disease	Zhang et al., 2020b,c	30 mice	Bilateral PC6	Qd, 20 min/time, pretreatment for 3 days	Rarefaction-dense wave, 2 Hz/15 Hz, 2 mA	Left ventricular ejection fraction of mice. The number of macrophages in spleen and heart of mice. Expression of NLRP3 and IL-1β in mouse myocardium.	EA pretreatment inhibits the activation of NLRP3 inflammasome
	Cai et al., 2014	60 mice	PC6 and BL15	Qd, 20 min/time, postconditioning for 3 days	2 Hz, 1 mA	Measurement of myocardial infarct size and ischemic area. Measure the expression of IL-10, IL-1β. Detect the protein expression of NF-κB p65.	EA postconditioning inhibits the NF-κB pathway.

EA, Electroacupuncture; IL, Interleukin; NF-κB, Nuclear factor-kappa B; NLRP3, NLR family pyrin domain containing 3.

### Reproductive system diseases

Primary dysmenorrhea (PD) is a painful menstrual spasm without any apparent pathology that occurs in up to 50% of women during menstruation, and increased secretion of vasoactive prostaglandin (PG) is the cause of PD (Dawood, 2006). Clinical experiments have shown that electroacupuncture can exert analgesic effect on PD patients (Song et al., 2015), but the research on the mechanism is still incomplete. Animal experiment showed that electroacupuncture stimulation at Guanyuan (RN4) and Sanyinjiao (SP6) can improve the pain symptoms and uterine pathological damage in PD rats, and the mechanism was related to the inhibition of the protein expression of NLRP3 and caspase-1, possibly because electroacupuncture inhibited the activation of NF-κB p65 and phospho-NF-κB p65, which are upstream of the NLRP3 inflammasome (Liu et al., 2019). The detailed experimental protocols are listed in Table 4.

**TABLE 4:** Mechanism of electroacupuncture targeting NLRP3 inflammasome in the treatment of primary dysmenorrhea.

Disease	Authors	Research object	Acupoints	Time	Frequency	Evaluation	Mechanism
Primary dysmenorrhea	Liu et al., 2019	50 mice	RN4, and SP6	Qd, 20 min/time, continuous for 10 days	Continuous wave, 50 Hz	Amount of body twisting in mice Pathological morphology and damage score of mice uterus NF-κB, phosphorylates NF-κB, NLRP3, caspase-1, IL-1β, and IL-18 in uterine tissue	EA inhibits the phosphorylation of NF-κB and activation of NLRP3 and caspase-1 in uterine tissues

EA, Electroacupuncture; IL, Interleukin; NLRP3, NLR family pyrin domain containing 3; NF-κB, Nuclear Factor-Kappa.

### Rheumatic immune system diseases

Gout is the most common cause of inflammatory arthritis worldwide (Hui et al., 2017). During the onset of acute gouty arthritis, monosodium urate crystals are formed by supersaturated uric acid concentrations in the joints, which act as foreign bodies and trigger the innate immune response, causing the immune system to overreact and leading to acute inflammatory reactions in the joints and surrounding tissues (Shi et al., 2013). Activation of the NLRP3 inflammasome and the release of IL-1β play key roles in the initiation of acute gout (Dalbeth et al., 2016). In animal experiments, Yu and colleagues reported that electroacupuncture stimulation of Neixiyan (EX-LE4) and Dubi (ST35) can reduce the inflammatory response of synovial tissue in the knee joint of rats, which may be related to the downregulation of NLRP3, ASC, caspase-1, IL-1β, and IL-18 expression, and reduce the occurrence of pyroptosis in synovial tissue (Yu et al., 2022). Similarly, in clinical trials, electroacupuncture alleviated the symptoms of joint pain, swelling and dysfunction by alleviating local inflammatory congestion and edema of the knee joint, and its mechanism was related to the inhibition of NLRP3/ASC/caspase-1 pathway, the downregulation of NLRP3 expression and inflammatory response transmitters, like TNF-α, 1L-1β (Zhang et al., 2020d). On the contrary, there are also clinical studies showing inconsistent conclusions that electroacupuncture may effectively upregulate the expression level of NLRP3 gene mRNA in patients and reduce serum levels of inflammatory factors to improve the clinical symptoms of gouty knee arthritis (Gao and Fu, 2019; Zhang Z. et al., 2019). However, the reason for this result is not clear, it may be related to the specific treatment of patients and may also be related to the NLRP3 gene polymorphism, the mechanism still needs to be further explored. Additionally, electroacupuncture stimulation of Sanyinjiao (SP6) and Zusanli (ST36) may inhibit NLRP3 inflammasome activation by reducing the activity of cathepsin B in the knee joint, thus treating acute gouty arthritis (Qiao et al., 2021). Consistently, it has been shown that NLRP3 inflammasome is inhibited in macrophages treated with cathepsin B chemical inhibitor (Hornung et al., 2008). The detailed experimental protocols are listed in Table 5.

**TABLE 5:** Mechanism of electroacupuncture targeting NLRP3 inflammasome in the treatment of gouty knee arthritis.

Disease	Authors	Research object	Acupoints	Time	Frequency	Evaluation	Mechanism
Gouty knee arthritis	Zhang et al., 2020d	106 patients with gouty knee arthritis	SP6, SP9, GB34, ST40, Gongsun (SP4), ST36, Ququan (LR8)	Qd, 40 min/time, continuous treatment for 1 week	Low frequency, rarefaction-dense wave	Measure the levels of TNF-α, IL-1β, IL-10, IL-4, CRP, ESR, UA. The expression of NLRP3, Caspase-1, ASC ET-1, MPO, sICAM-1, NO SF-36 scale	EA reduces inflammatory response transmitters by inhibiting NLRP3/ASC/Caspase-1 pathway.
	Qiao et al., 2021	60 mice	SP6 and ST36	Qd, 10 min/time, continuous for 1 week	rarefaction-dense wave, 2/100 Hz, 1 mA	Score the gait of rats. Observe the pathological morphology of synovial tissue. Measure the levels of cathepsin- B protein, NLRP3, ASC, caspase-1, IL-1β, and IL-18.	EA may inhibit the cathepsin-B/NLRP3 pathway

ASC, Apoptosis-associated speck-like protein; CRP, C-reactive protein; EA, Electroacupuncture; ESR, Erythrocyte Sedimentation Rate; ET-1, Endothelin-1; IL, Interleukin; NLRP3, NLR family pyrin domain containing 3; TNF-α, Tumor Necrosis Factor Alpha; UA, Uric acid; MPO, Myeloperoxidase; NO, Nitric Oxide; sICAM-1, Soluble intercellular adhesion molecule-1.

### Other diseases

Depression is a common disease, affecting approximately 280 million people worldwide (World Health Organization [WHO], and Depression, 2021). At its worst, depression can lead to suicide, with more than 700,000 people dying from suicide each year. The pathogenesis of depression is complex, and current studies have shown that it is closely related to inflammatory reaction, monoamine neurotransmitters and HPA endocrine axis (Gao et al., 2021). NLRP3 has been shown to play an important role in the pathogenesis of depression, acting as a key molecule regulating the activation of the immune inflammatory response and depression (Feng et al., 2019). Acupuncture stimulation at Baihui (GV20) and Yintang (EX-HN3) may inhibit the expression of NLRP3, ASC and caspase-1, which are the key NLRP3 inflammasome molecules in the prefrontal cortex, thus reducing the inflammatory response and thereby improving depression (Wang et al., 2020). Yue and colleagues reported consistent responses to electroacupuncture stimulation at Baihui (GV20) and Yanglingquan (GB34) (Yue et al., 2018). In addition, electroacupuncture stimulation at the auricular concha can improve depression, possibly by inhibiting the activation of NF-κB, an upstream factor of NLRP3 in the prefrontal cortex, and thus inhibiting inflammation in the central nervous system (Liu Y. et al., 2020).

Inflammatory pain is nerve stimulation caused by injury-related chemicals. Electroacupuncture has a good analgesic effect, and its analgesic mechanism is a variety of bioactive molecules in the process of pain, but the peripheral and central analgesic mechanisms mediated by acupuncture are still under constant study (Li et al., 2018). Previous studies have shown that electroacupuncture regulated inflammatory response by inhibiting the activation of NLRP3 inflammasome and the expression of inflammatory factors. Electroacupuncture stimulation at Huantiao (GB30) and Yanglingquan (GB34) could inhibit NLRP3 inflammasome activation and delay the maturation of IL-1β by activating the peripheral cannabinoid (CB2) receptor and improving inflammatory pain (Gao et al., 2018). In addition, electroacupuncture promotes Treg cells, induces the production of the anti-inflammatory cytokine IL-10, inhibits macrophages and neutrophils, reduces NLRP3 expression, and relieves inflammatory pain (Yu et al., 2020).

Dry eye is a common ophthalmic disease, and its occurrence and development are related to various factors. Inflammation is considered to be the main cause. Clinical research showed that the effective rate of acupuncture in the treatment of dry eye was 38.3% higher than that of sodium hyaluronate eye drops (Zhu et al., 2019). Acupuncture can promote lacrimal gland secretion and stabilize tear film, but its underlying mechanism has not been revealed. Yang and colleagues reported that electroacupuncture increased tear production, prolonged break-up time (BUT), and enhanced tear film stability, mainly by inhibiting the expression of ROS, thioredoxin-interacting protein (TXNIP), and NLRP3 inflammatory to improve dry eye syndrome (Yang et al., 2022). The detailed experimental protocols are listed in Table 6.

**TABLE 6:** Mechanism of electroacupuncture targeting NLRP3 inflammasome in the treatment of depression, inflammatory pain, and dry eye syndrome.

Disease	Authors	Research object	Acupoint	Time	Frequency	Evaluation	Mechanism
Depression	Yue et al., 2018	33 mice	GV20 and GB34 (the right side)	Qod, 30 min/time, continuous for 4 weeks	2/100 Hz, 0.3 mA	Open field test and forced swimming test. Measure the levels of NLRP3, ASC, caspase-1, IL-1β.	EA inhibits the expression of NLRP3 inflammasome
	Liu Y. et al., 2020	24 mice	Auricular concha	Qd, 30 min/time, continuous for 3 weeks	Rarefaction-dense wave, 2/15 Hz	Open field test and sugar water preference experiment. Measure the levels of NLRP3, NF-κB, and IL-1β.	EA inhibits the NF-κB/NLRP3 pathway
Inflammatory pain	Gao et al., 2018	96 mice	GB30 and GB34	Qod, 30 min/time, 3 times in total	2 Hz	Activity of caspase-1. Macrophages, T cells, keratinocytes, and NLRP3.	EA inhibits the activation of NLRP3 inflammasomes by stimulating CB2 receptor
	Yu et al., 2020	CFA-induced mice (quantity not indicated)	ST36 and SP6	Qd, 20 min/time, continuous 5 days	2/100 Hz, 2 mA	Dynamic Plantar Aesthesiometer and Plantar test. Measure the expression of IL-10, IL-1β, NLRP3, and TNF-α. Measure the levels of macrophages, neutrophil, and Treg cells in spleen tissue.	EA inhibits the expression of NLRP3, IL-1β, and TNF-α
Dry eye syndrome	Yang et al., 2022	50 mice	Bilateral TE23, GB20, and GB37	Qd, 20 min/time, continuous 7 days	Continuous waveform, 2 Hz, 1–2 mA	Measure the ROS activity. Examine the mRNA expression of TXNIP, NLRP3, ASC and caspase-1. Detect the protein expression of TXNIP, NLRP3, IL-18, IL-1β. Observe the tear secretion and tear film break-up time.	EA inhibits the ROS/TXNIP/NLRP3 signaling pathway

ASC, Apoptosis-associated speck-like protein; CFA, Complete Freund’s adjuvant; EA, Electroacupuncture; IL, Interleukin; NLRP3, NLR family pyrin domain containing 3; NF-κB, Nuclear Factor-Kappa B; ROS, Reactive Oxygen Species; TXNIP, Thioredoxin-interacting Protein.

## Discussion

Although there are various ways to treat diseases in clinic, such as drug therapy and surgical treatment, acupuncture is widely concerned because of its simple, convenient, safe and effective characteristics when drug intervention has large adverse reactions or single treatment effect is not significant, or the disease or symptoms need to be combined with adjuvant therapy. At present, more and more clinical studies have reported acupuncture in the treatment of various diseases. It has been pointed out that acupuncture can effectively regulate the spasm of bladder detrusor and urethral sphincter and promote the excretion of urine for urinary retention after spinal cord injury (Zhang C. et al., 2019). However, electroacupuncture uses electrical stimulation with different frequencies and intensities, which can stimulate acupoints more than twisting needles or other manual manipulation techniques. It makes up for the shortcomings of acupuncture with insufficient stimulation intensity and shows better efficacy in clinical studies. Studies have shown that, compared with acupuncture, electroacupuncture has better effects in alleviating local pain (Aranha et al., 2015), regulating lipid metabolism in patients with non-alcoholic fatty liver disease, reducing inflammatory reaction (Yu et al., 2018), promoting lacrimal gland secretion and stabilizing tear film (Zhang et al., 2020a). However, the research on the internal mechanism of electroacupuncture is not comprehensive. This article reviews the mechanism of NLRP3 inflammasome in electroacupuncture, which provides convenience and direction for researchers to conduct double-blind randomized controlled experiments and obtain good clinical evidence. It provides certain evidence support for the future use of electroacupuncture as a treatment in a wide range of clinical practice.

**FIGURE 1:**
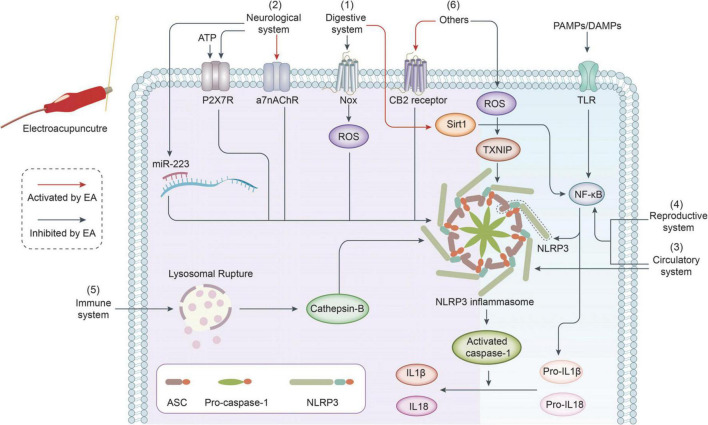
Summary of mechanisms of electroacupuncture targeting NLRP3 inflammasome in treatment of various diseases. The common point is that electroacupuncture can treat diseases by inhibiting the expression of NLRP3, thereby down-regulating the expression of pro-inflammatory factors IL-18 and IL-1β (Shi et al., 2020). Digestive system: electroacupuncture inhibits NOX/ROS/NLRP3 pathway; electroacupuncture inhibits NF-κB/NLRP3 pathway by enhancing the expression of Sirt1 (Xie et al., 2021). Neurological system: electroacupuncture inhibits ATP/P2X7R/NLRP3 and miR-223/NLRP3 pathway; electroacupuncture inhibits NLRP3 inflammasome by enhancing the expression of a7nAChR (Meng et al., 2018). Circulatory system: electroacupuncture inhibits the activation of NLRP3 inflammasome by down-regulating the expression of NLRP3, ASC, and pro-caspase-1; electroacupuncture inhibits NF-κB/NLRP3 pathway (Zhu et al., 2015). Reproductive system: electroacupuncture inhibits NF-κB/NLRP3 pathway (Zhang et al., 2017). Immune system: electroacupuncture inhibits NLRP3 inflammasome by down-regulating the expression of Cathepsin-B (Zhang et al., 2020a). Others: electroacupuncture inhibits ROS/TXNIP/NLRP3 pathway; electroacupuncture inhibits NLRP3 inflammasome by up-regulating CB2 expression.

According to a literature review, the conclusion of all studies is almost the same, namely, electroacupuncture can inhibit the activation or expression of NLRP3 inflammasome through a variety of different pathways, thus achieving therapeutic effects in various diseases (Figure 1). It is not difficult to find that almost all mechanisms in the reviewed articles have common points, suggesting that electroacupuncture targets NLRP3 to treat diseases mainly by reducing the inflammatory response. Electroacupuncture reduces inflammation and symptoms by inhibiting NLRP3 inflammasome activation and the secretion of proinflammatory factors, such as IL-1β. These mechanisms include upstream signaling pathways that inhibit NLRP3 regulation, such as the NF-κB/NLRP3, ATP/P2X7R/NLRP3, and ROS/TXNIP/NLRP3 signaling pathways. NLRP3 activation is also reduced by inhibiting NLRP3, ASC, and Caspase-1 protein expression. Therefore, the mechanism by which electroacupuncture targets the NLRP3 inflammasome in the treatment of diseases may involve the downregulation of NLRP3 inflammasome expression and inhibition of the inflammatory response.

Existing studies were all carried out *in vivo*, and the experimental subjects were mainly rat models. Only two studies were conducted on humans (Gao and Fu, 2019; Zhang et al., 2020d), both of which focused on gouty knee arthritis. Most of the studies concluded that electroacupuncture inhibited NLRP3 inflammasome activation; however, the level of evidence differed, and two studies (Liu et al., 2019; Liu L. et al., 2021) used drug therapy as a control group, while one study used receptor CB2 knockdown rats (Qiao et al., 2021) to enhance the level of evidence. The other studies used a blank group, sham operation group and/or sham acupuncture group as control groups, and the level of evidence needs to be strengthened.

Studies based on rat models used different intervention protocols, such as the electroacupuncture points, frequency, duration of retention, and duration of the electroacupuncture course. It is not clear whether the efficacy of electroacupuncture at different points, currents and frequencies is consistent. According to the literature, the acupoints of Baihui (GV20), Zusanli (ST36), and Sanyinjiao (SP6) were selected with relatively high frequency. Moreover, two studies (Song et al., 2019; Hou et al., 2020) showed that high-frequency electroacupuncture was more effective than low-frequency electroacupuncture, triggering different signaling pathways and enhancing the inhibitory effect on the NLRP3 inflammasome. Relatively consistently, the needle retention time mentioned in different experimental protocols was mostly 20 or 30 min/time. The duration of electroacupuncture varied from 3 to 56 days, and the fluctuation range was wide. It is not clear whether the treatment effect was significant over time. A handful of studies (Guo et al., 2021; Liu L. et al., 2021; Mei et al., 2021) comparing the effects of different treatment durations showed that 7 and 21 days of treatment were better than 1 and 3 days of treatment, and there was reduced expression of NLRP3, ASC, and caspase-1 and reduced secretion levels of IL-1β. However, the results did not indicate whether the effects of 7 and 21 days of treatment on NLRP3 inflammasome expression were statistically significant.

## Conclusion

In summary, the authors found that the NLRP3 inflammasome plays an important role in the treatment of some diseases by electroacupuncture. The main mechanism is that electroacupuncture reduces the inflammatory response through the NLRP3 inflammasome. However, the existing studies focus on animal experiments, and we should also pay attention to clinical trials in the future. The protocol of effective electroacupuncture treatment for gouty knee arthritis can be applied to the treatment of other diseases. Advanced technologies such as high-throughput sequencing and single-cell sequencing can be used to extract clinical samples for further mechanism research. Second, researchers can enhance the level of evidence by using NLRP3 inhibitors or agonists in experiments. For example, MCC950 can block ASC accumulation and inhibit both typical and atypical NLRP3 inflammasomes (Shao et al., 2015) as well as NLRP3 gene knockout mice. Furthermore, in the future, electroacupuncture treatment protocols should be unified, such as the electroacupuncture point, frequency, duration of needle retention and duration of treatment course, to observe whether there is a difference in the effects of different experimental protocols on the expression of the NLRP3 inflammasome. Finally, current studies mostly focus on electroacupuncture rather than the NLRP3 inflammasome, which inhibits neuroinflammation. The internal mechanism of action by which pathway to inhibit NLRP3 inflammasome activation or expression is not complete, and further research is needed. If we can use randomized double-blind control to verify the effectiveness of electroacupuncture in the treatment of diseases, it will promote the development of human health.

## Author contributions

HL conceived and supervised the study. JY drew the figure. DW and MY wrote the manuscript. All authors reviewed the results and approved the final version of the manuscript.
